# Clinicopathological and Prognostic Features of Pleomorphic Adenoma, Recurrent Pleomorphic Adenoma, and Carcinoma ex Pleomorphic Adenoma: A 20‐Year Retrospective Analysis

**DOI:** 10.1111/odi.70261

**Published:** 2026-03-17

**Authors:** Talita de Carvalho Kimura, Luccas Lavareze, Reydson Alcides de Lima‐Souza, Nívia Castro Binda, Carlos Takahiro Chone, Alfio José Tincani, Albina Altemani, Fernanda Viviane Mariano

**Affiliations:** ^1^ Department of Pathology, School of Medical Sciences University of Campinas (UNICAMP) Campinas Brazil; ^2^ Department of Oral Diagnosis, Piracicaba Dental School University of Campinas (UNICAMP) Piracicaba Brazil; ^3^ Department of Ophthalmology and Otorhinolaryngology, School of Medical Sciences University of Campinas (UNICAMP) Campinas Brazil; ^4^ Department of Surgery, Head and Neck Surgery, School of Medical Sciences University of Campinas (UNICAMP) Campinas Brazil

**Keywords:** carcinoma ex pleomorphic adenoma, histopathology, pleomorphic adenoma, prognostic factors, recurrent pleomorphic adenoma, salivary gland tumors, survival analysis

## Abstract

**Background:**

Pleomorphic adenoma (PA) is the most common salivary gland tumor, and a subset may recur as recurrent PA (RPA) or progress to carcinoma ex pleomorphic adenoma (CXPA). However, comparative evaluations of their biological behavior and prognostic factors remain limited.

**Objective:**

To characterize and compare the clinical features, pathological patterns, treatment outcomes, and prognostic factors of PA, RPA, and CXPA over a 20‐year period at a Brazilian tertiary referral center.

**Materials and Methods:**

A retrospective review of 234 cases (148 PA, 23 RPA, 63 CXPA) diagnosed between 2004 and 2024. Analyses included Fisher's exact test and chi‐square test for group comparisons and Cox proportional hazards models for overall (OS) and disease‐specific survival (DSS).

**Results:**

PA and RPA mainly affected younger females and presented smaller tumors and shorter clinical evolution than CXPA (*p* < 0.01). In CXPA, perineural invasion, angiolymphatic invasion, necrosis, recurrence, symptomatology, distant metastasis, and capsular invasion pattern were associated with reduced OS and DSS (*p* < 0.05).

**Conclusion:**

This retrospective study demonstrates distinct clinical and pathological profiles across PA, RPA, and CXPA. In CXPA, clinical and histological factors strongly predict survival and should guide risk stratification, therapeutic decisions, and follow‐up strategies.

## 1 Introduction

Salivary gland tumors are a heterogeneous group of neoplasms that include both benign and malignant lesions, accounting for approximately 3%–5% of all head and neck neoplasms (Tian et al. 2010). Benign tumors are the most prevalent, comprising approximately 50%–80% of cases (da Silva et al. 2018), with pleomorphic adenoma (PA) being the most common in this location, accounting for 50%–70% of all cases (Sando et al. 2016; Andreasen et al. 2016; Valstar et al. 2017; Cunha et al. 2021). PA predominantly arises in the parotid gland and usually presents as a slowly growing mass that occurs across a wide age range. Peak incidence occurs in the fifth and sixth decades of life, and there is a slight female predilection (Andreasen et al. 2016; Valstar et al. 2017). Histologically, PA is characterized by cytomorphological and architectural diversity, comprising epithelial and myoepithelial cells, which are typically embedded in a stromal component that is fibrous, myxoid or chondroid tissue (de Sousa Lopes et al. 2017).

Although complete surgical resection is curative and the treatment of choice for PA, recurrences may occur in 2.9%–6.7% of cases, primarily as a result of rupture and incomplete tumor excision (Zbären et al. 2005; Andreasen et al. 2016; Valstar et al. 2017). Recurrent PA (RPA) typically presents as a multinodular disease, with histopathological features characterized by multiple well‐defined PA nodules separated by a fibrous tissue, embedded within residual salivary gland tissue (Zbären et al. 2005; Witt et al. 2015). Over time, pleomorphic adenomas (PAs) may undergo malignant transformation into carcinoma ex pleomorphic adenoma (CXPA), especially when tumors are recurrent, persistent, or long‐standing. The literature estimates that the rate of malignant transformation is 3% in PA and about 6% in RPA (Di Palma 2013; Andreasen et al. 2016; Valstar et al. 2017).

CXPA is a malignancy of epithelial and/or myoepithelial origin arising from either a primary or recurrent PA. It accounts for approximately 3.6% of all salivary gland tumors and 12% of salivary gland malignancies (Katabi et al. 2010; Griffith et al. 2014). Similar to PA, CXPA predominantly affects women and typically occurs in older individuals, with peak incidence in the sixth and seventh decades of life (Weiler et al. 2011; Mariano et al. 2013). Histologically, the carcinomatous component may vary into epithelial and/or myoepithelial phenotype, with salivary duct carcinoma (SDC) being the most common, followed by myoepithelial carcinoma (MC) (Weiler et al. 2011; Mariano et al. 2013). In CXPA, the PA component may exhibit variable cellularity and can present either intermingled with the carcinoma or as a distinct nodule, often hyalinized and, in rare cases, entirely sclerotic (de Lima‐Souza et al. 2021). In cases where the benign portion is absent, previous clinicopathological documentation of PA at the same site is required the diagnosis (Mariano et al. 2013). CXPA is also classified based on the extent of invasion beyond the PA capsule into intracapsular (IC), minimally invasive (MI), and invasive/frankly invasive (FI) (Mariano et al. 2013). The primary treatment for CXPA is wide local excision, often combined with adjuvant radiotherapy for invasive tumors or those with positive margins. The 5‐year survival rate for CXPA varies widely, ranging from 25% to 75%, depending on the extent of the disease and the treatment modality (Lewis et al. 2001; Katabi et al. 2010; Weiler et al. 2011; Mariano et al. 2013; Gupta et al. 2019).

While PA, RPA, and CXPA are well‐established in the literature, most clinicopathological studies have focused on PA and CXPA separately or on selected aspects of malignant transformation. Simultaneous comparative analyses of PA, RPA, and CXPA within a single institutional cohort remain scarce, particularly with respect to clinicopathological features, treatment outcomes, and prognostic factors. In this context, the present study aimed primarily to compare the clinicopathological characteristics and treatment outcomes of PA, RPA, CXPA cases diagnosed at a single Brazilian tertiary referral center. Secondarily, this study sought to identify clinicopathological prognostic factors associated with overall survival (OS) and disease‐specific survival (DSS) in patients with CXPA, contributing data from a Latin American population.

## 2 Materials and Methods

### 2.1 Case Selection

This is a retrospective cross‐sectional study that analyzed clinicopathological data from a large sample of PA, RPA and CXPA cases diagnosed between 2004 and 2024. The cases were collected from a Brazilian tertiary hospital (Clinical Hospital of the University of Campinas) using the archives of the Department of Pathology of the School of Medical Sciences, University of Campinas. This study was submitted and approved by the Research Ethics Committee of the University of Campinas (UNICAMP) (CAAE: 59408016.7.0000.5404; CAAE: 59408016.7.3001.5418).

All cases initially identified during the study period were reviewed, and those not arising in major or minor salivary glands, with incomplete medical records, or with insufficient histological material for diagnostic confirmation were excluded. The final study cohort comprised 148 PA, 23 RPA, and 63 CXPA cases. RPA was defined as a histologically confirmed PA occurring at the same anatomical site as a previously excised PA, with documented clinical or pathological evidence of recurrence.

It is important to note that the PA, RPA, and CXPA cases included in this study represent independent diagnostic groups and were not derived from longitudinal follow‐up of the same patients. Thus, the analysis does not reflect a progression sequence from PA to RPA or CXPA, but rather a cross‐sectional comparison of distinct tumor categories. In addition, of the 63 CXPA cases included in this study, 38 had been previously reported by our group in an earlier publication (Mariano et al. 2013). Although some cases have been reported in previous publications, the present investigation substantially expands upon that prior report by incorporating additional clinicopathological variables, a comparative analysis including PA and RPA cases, and a systematic evaluation of prognostic factors. Importantly, the survival analyses and clinicopathological prognostic associations presented here were not explored in the previous publication, thereby providing novel insights into the clinical behavior of CXPA.

### 2.2 Clinicopathological Analysis

All PA, RPA, and CXPA cases were microscopically reviewed by at least two experienced oral and maxillofacial pathologists to confirm the diagnosis based on the criteria set by the latest version of the World Health Organization (WHO) Classification for Head and Neck Tumors (5th edition). In cases of discrepant interpretation, the slides were jointly reviewed to reach a consensus diagnosis. Formal interobserver agreement statistics were not calculated. Patient information, including clinical and demographic data, was retrieved from the medical records. This included sex, age, anatomical location, lesion size, clinical presentation, duration, symptomatology, imaging characteristics, histopathological characteristics and diagnosis, TNM (T, extent of the tumor; N, spread to lymph nodes; M, metastasis) and clinical stage, treatment modalities, follow‐up, recurrences, metastasis, and survival status. Any missing data were reported as not available (NA).

For the histopathological analysis, the cases were reviewed using 3‐μm hematoxylin–eosin (H&E) stained sections of formalin‐fixed paraffin‐embedded samples. In PA and RPA cases, the following characteristics were evaluated: capsule infiltration, ductal formation, stroma, increased mitotic activity, necrotic foci, differentiations (squamous, osseous, sebaceous). Tumors were also categorized based on the relative proportion of epithelial and stromal components; tumors were categorized into three subtypes: classic (balanced stroma and cellularity), stroma‐rich (predominantly myxoid or chondroid matrix), and cell‐rich (high epithelial density with reduced stroma). In CXPA cases, tumors were classified according to histological subtype and capsular extension as IC, MI (≤ 1.5 mm through capsule), or FI (> 1.5 mm through capsule) (Katabi et al. 2010).

For patients with RPA, the age at initial PA diagnosis was not directly available in all cases. Therefore, an estimated age at primary PA diagnosis was calculated by subtracting the documented time to recurrence from the age at RPA diagnosis. This variable was used for exploratory comparative analyses and should be interpreted with caution, as it assumes accurate documentation of time to recurrence and may be subject to recall or recording bias inherent to retrospective studies.

### 2.3 Statistical Analysis

A descriptive analysis was conducted, with categorical variables presented as absolute values and percentages, and continuous variables as means, standard deviation (SD), and range. The association between independent variables (clinicopathological variables) and tumor type (PA vs. RPA) and (benign (PA and RPA) vs. malignant) was assessed using Fisher's exact test or the chi‐square test, as appropriate using GraphPad Prism (v. 10.4.0). For these comparative analyses, continuous variables were categorized using predefined cutoffs: tumor size was dichotomized as < 4.0 cm versus ≥ 4.0 cm, and clinical evolution time was categorized as ≤ 45 months versus > 45 months, based on clinically relevant thresholds and the median values observed in the study population.

OS was defined as the time from the date of histopathological diagnosis to death from any cause or last follow‐up. DSS was defined as the time from diagnosis to death attributable to CXPA or last follow‐up. Patients who were alive at last follow‐up or lost to follow‐up were censored at the date of last documented clinical contact. Only patients with adequate follow‐up and well‐documented outcomes were included in the survival analyses. Survival outcomes were evaluated using univariate Cox proportional hazards regression to identify clinicopathological factors associated with OS and DSS in CXPA. Multivariate Cox regression was not performed due to the limited number of outcome events, which would have violated the events‐per‐variable rule and increased the risk of model overfitting, potentially leading to unstable or unreliable estimates. A *p*‐value < 0.05 was considered statistically significant. Statistical analyses were performed using R (version 4.4.0) with the “survival” package (v. 3.5–8), and data visualization was conducted using GraphPad Prism (v. 10.4.0).

Missing data were handled using a pairwise deletion approach. For each analysis, only cases with available information for the variables under evaluation were included, and cases with missing data were excluded from that specific test but retained for other analyses when applicable. No data imputation was performed.

## 3 Results

### 3.1 Descriptive Analysis

Clinical and pathological characteristics of the PA, RPA, and CXPA groups are summarized in Table 1, including age, sex, tumor site, clinical presentation, tumor size, symptom duration, treatment, and follow‐up outcomes.

**TABLE 1:** Summary of clinicopathological features of pleomorphic adenoma, recurrent pleomorphic adenoma, and carcinoma ex pleomorphic adenoma.

Variables	PA (*n* = 148)	RPA (*n* = 23)	CXPA (*n* = 63)
	Count (percentage %)		
Sex			
Female	85 (57.4%)	17 (75.0%)	23 (36.5%)
Male	63 (42.6%)	6 (25.0%)	35 (55.6%)
Not informed	—	—	5 (7.9%)
Location			
Parotid	119 (80.4%)	23 (100.0%)	44 (69.8%)
Submandibular	19 (12.8%)	—	9 (14.3%)
Minor salivary gland	10 (6.8%)	—	9 (14.3%)
Smoking habit			
No	95 (64.2%)	16 (69.6%)	12 (19.0%)
Yes	53 (35.8%)	7 (30.4%)	19 (30.2%)
Not informed	—	—	32 (50.8%)
Alcohol consumption			
No	136 (91.9%)	20 (87.5%)	19 (30.2%)
Yes	12 (8.1%)	3 (13.0%)	12 (19.0%)
Not informed	—	—	32 (50.8%)
Symptoms			
Asymptomatic	125 (84.5%)	19 (82.6%)	20 (31.7%)
Painful	12 (8.1%)	4 (17.4%)	6 (9.5%)
Not informed	11 (7.4%)	—	35 (55.6%)
Clinical presentation			
Fibroelastic nodule	146 (98.6%)	14 (60.9%)	32 (50.8%)
Multinodular fibroelastic nodule	—	9 (39.1%)	—
Not informed	2 (1.4%)	—	31 (49.2%)
Fixation			
Absent	89 (60.5%)	21 (91.3%)	12 (19.0%)
Present	24 (16.3%)	2 (8.7%)	5 (7.9%)
Not informed	34 (23.1%)	—	46 (73.0%)
Size			
Range	0.5–12.0 cm	1.0–6.0 cm	1.5–10.0 cm
Mean	2.53 cm	3.14 cm	5.06 cm
Standard deviation	1.33 cm	1.43 cm	2.24 cm
Clinical evolution time			
Range	1–456 months	3–156 months	3–360 months
Mean	45.8 months	45.9 months	98.0 months
Standard deviation	65.5 months	47.0 months	100.0 months
Treatment			
Surgery alone	134 (90.5%)	23 (100.0%)	30 (47.6%)
Surgery + neck dissection	9 (6.1%)	—	4 (6.3%)
Surgery + radiotherapy	4 (2.7%)	—	13 (20.6%)
Surgery + radiotherapy + chemotherapy	1 (0.7%)	—	—
Surgery + neck dissection + radiotherapy	—	—	9 (14.3%)
Surgery + neck dissection + chemotherapy	—	—	1 (1.6%)
Not informed	—	—	6 (9.5%)
Surgical margins			
Positive	74 (50.3%)	5 (21.7%)	21 (33.3%)
Negative	61 (41.5%)	3 (13.0%)	23 (36.5%)
Not available	12 (8.2%)	15 (65.3%)	19 (30.2%)
Histological subtype			
Adenocarcinoma, NOS	—	—	20 (31.7%)
Epidermoid carcinoma	—	—	1 (1.6%)
Epithelial–Myoepithelial carcinoma	—	—	9 (14.3%)
Myoepithelial carcinoma	—	—	9 (14.3%)
Sarcomatoid carcinoma	—	—	1 (1.6%)
Salivary duct carcinoma	—	—	22 (34.9%)
Secretory carcinoma	—	—	1 (1.6%)
Capsular invasion			
Intracapsular	—	—	17 (27.0%)
Minimally invasive	—	—	15 (23.8%)
Invasive/Frankly invasive	—	—	31 (49.2%)
Follow‐up			
Range	3.0–182 months	3.6–182.9 months	0.3–371.9 months
Mean	31.9 months	78.7 months	57.6 months
Standard deviation	36.5 months	53.1 months	69.9 months
Outcome			
NED	143 (96.6%)	23 (100.0%)	21 (33.3%)
DOD	—	—	11 (17.5%)
DOC	3 (2.0%)	—	5 (7.9%)
LFU	2 (1.4%)	—	26 (41.3%)

Abbreviations: CXPA, carcinoma ex pleomorphic adenoma; DOC, died of other causes; DOD, died of disease; LFU, lost to follow‐up; NED, no evidence of disease; NOS, not otherwise specified; PA, pleomorphic adenoma; RPA, recurrent pleomorphic adenoma.

### 3.2 Pleomorphic Adenoma

A total of 148 cases of PA were included in this study. The mean age at diagnosis was 43.7 years (SD: 15.9; range: 8–83 years). There was a slight predominance of female patients, with 85 women (57.4%) and 63 men (42.6%). Most tumors were in the parotid gland (119 cases; 80.4%), followed by the submandibular gland (19 cases; 12.8%) and minor salivary glands (10 cases; 6.8%). Regarding lifestyle factors, 95 patients (64.2%) were non‐smokers and 12 (8.1%) reported alcohol consumption.

Clinically, most tumors were asymptomatic at diagnosis (125 cases; 84.5%) and exhibited a mobile (89 cases; 60.5%) fibroelastic nodule (146 cases; 98.6%). Tumor size ranged from 0.5 to 12.0 cm, with a mean of 2.53 ± 1.33 cm. The time from symptom onset to diagnosis ranged from 1 to 456 months, with a mean of 45.8 ± 65.5 months. Imaging by ultrasound was available in 113 cases. The most frequent finding was a solid, hypoechoic, well‐defined nodule (66 cases; 45.5%).

**FIGURE 1:**
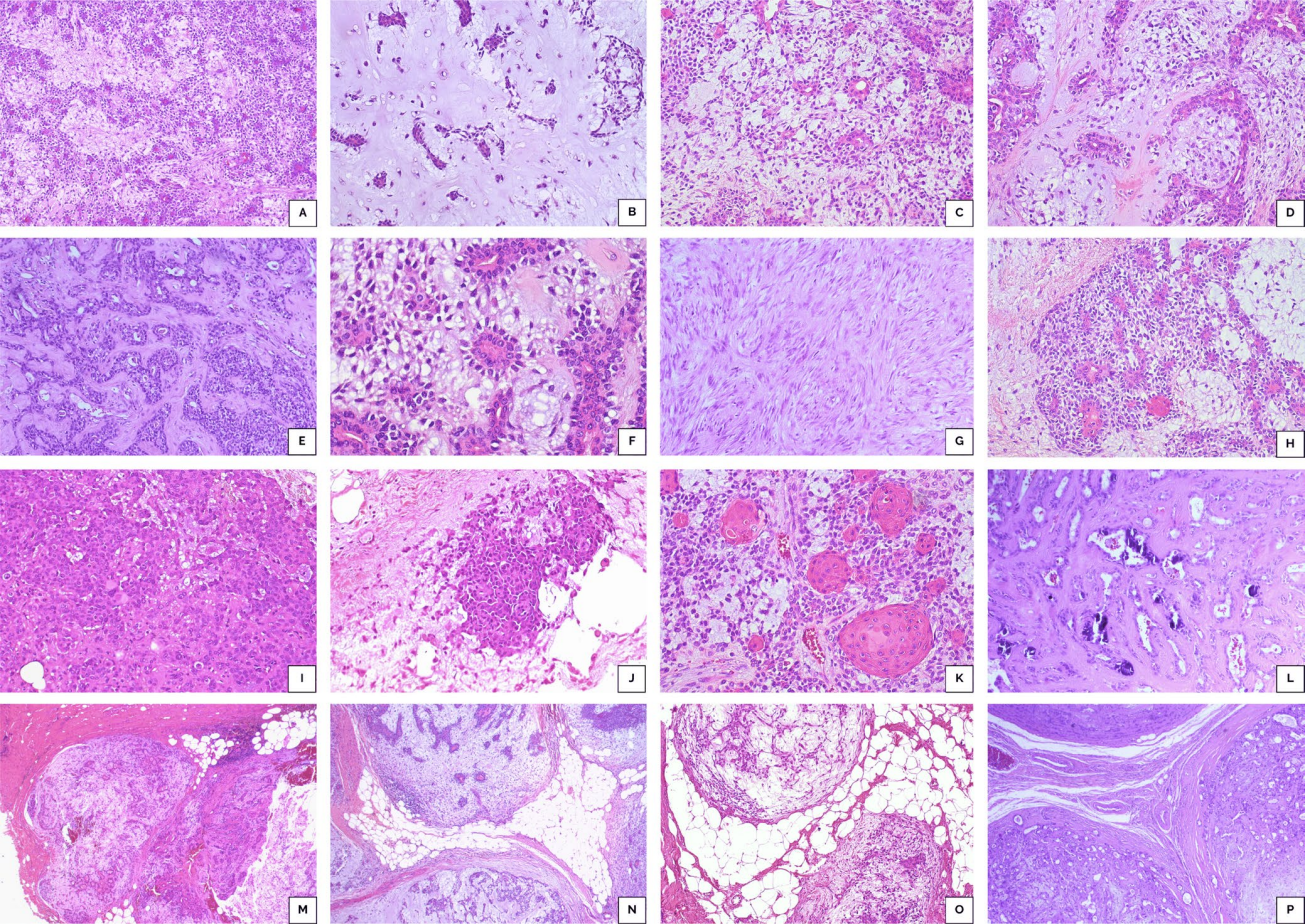
Representative histopathological features of pleomorphic adenoma (PA) and recurrent pleomorphic adenoma (RPA). (A–E) Stromal heterogeneity observed in PA: (A) classical biphasic architecture with ductal epithelial and myoepithelial components embedded in a fibromyxoid matrix (10×); (B) mature chondroid stroma (20×); (C) myxoid stroma (20×); (D) chondromyxoid matrix with alternating basophilic and eosinophilic zones (20×); (E) densely fibrous and hyalinized stroma (20×). (F) Prominent ductal structures lined by eosinophilic epithelial cells and pale, elongated myoepithelial cells (40×). (G–J) Spectrum of myoepithelial cell morphologies: (G) spindle‐shaped cells (20×); (H) clear‐cell phenotype (20×); (I) epithelioid cells with prominent borders (20×); (J) plasmacytoid cells with eccentric nuclei and eosinophilic cytoplasm (20×). (K) Squamous metaplasia with focal keratin pearl formation (20×). (L) Dystrophic calcifications within the stromal compartment (10×). (M) Tumor nodule demonstrating focal capsular invasion (5×). (N–P) Multinodular growth pattern characteristic of RPA, with multiple non‐encapsulated nodules separated by fibrous septa, often embedded in fibrotic or scarred salivary tissue (5× each). All sections were stained with hematoxylin and eosin (H&E).

Histologically, PA displays a wide diversity of morphologic features and growth patterns. PAs normally exhibit biphasic histological architecture, composed of epithelial and myoepithelial elements arranged in ductal structures, solid nests, or cords (Figure 1). Among the 89 cases analyzed, abundant ductal formation was the most frequent epithelial arrangement (35 cases; 39.3%), while myxoid or myxoid/hyalinized stroma represented the most common stromal subtype (50 cases; 56.2%). The tumors were generally encapsulated, with complete capsules observed more frequently in major salivary glands and partial or incomplete capsules noted in tumors arising from minor salivary glands.

The cellular morphology was heterogeneous. Most cells displayed round, angular, or oval shapes. Less frequently, plasmacytoid, spindle‐shaped, cuboidal, polygonal, oncocytic, basal‐like, and mucous cell morphologies were observed. Tubular‐like as well as cribriform‐like, and basal cell adenoma–like patterns, were present in variable proportions. Sebaceous differentiation was also identified in a subset of cases. Cystic degeneration and areas of squamous metaplasia were additional findings. Necrosis was generally absent, identified in only two cases. Mitotic figures were rare. In a minor proportion of cases (8 cases), focal and mild cytological pleomorphism was observed. Among the subtypes, the classic type was the most common (44 cases, 49.4%), followed by stroma‐rich (28 cases, 31.5%) and cell‐rich (17 cases, 19.1%).

Regarding treatment, all patients underwent surgical management, whereas 134 (90.5%) received surgery alone, 9 (6.1%) underwent surgery with neck dissection, 4 (2.7%) received surgery followed by radiotherapy, and 1 patient (0.7%) received multimodal treatment including surgery, radiotherapy, and chemotherapy. This multimodal approach was indicated in a patient with a large parotid PA causing tracheal compression and exhibiting positive surgical margins after the surgery. For tumors located in the parotid gland, the most common procedure was superficial parotidectomy (100 cases; 67.6%). Surgical margins were compromised in 74 cases (50.3%) and free in 61 (41.5%). Follow‐up data were available for 136 patients, with a mean follow‐up duration of 31.9 months (SD: 36.5 months). At last follow‐up, 143 patients (96.6%) were alive without evidence of disease, 3 (2.0%) had died of other causes, and 2 (1.4%) were lost to follow‐up.

### 3.3 Recurrent Pleomorphic Adenoma

A total of 23 cases of RPA were included. The mean age at diagnosis was 46.2 years (SD: 12.1; range: 19–68 years). Female patients were more frequently affected (17 cases; 75.0%), compared to males (6 cases; 25.0%). All cases occurred in the parotid gland, with no involvement of minor salivary glands. A history of smoking was reported in 7 patients, and alcohol consumption was reported in 3 patients (13.0%).

Clinically, most lesions presented as mobile (21 cases; 91.3%), asymptomatic (19 cases; 82.6%), fibroelastic nodules (14 cases; 60.9%), or multiple fibroelastic nodules (9 cases; 39.1%). Tumor size ranged from 1.0 to 6.0 cm, with a mean of 3.14 ± 1.43 cm. The time from symptom onset to diagnosis ranged widely from 3 to 156 months, with a mean of 45.9 ± 47.0 months. Time to recurrence ranged from 3 to 360 months. Most patients (17 cases; 73.9%) had a single recurrence, while 5 (21.7%) experienced two recurrences and 1 (4.3%) had three.

Histologically, RPA exhibited architectural and cytological features like those observed in primary PAs, with epithelial and myoepithelial elements arranged in duct‐like structures, cords, or solid nests. The stroma was variably fibrous, hyalinized, or myxoid, with occasional chondroid areas. However, distinct morphological patterns were noted in the recurrent setting. Most cases demonstrated a multinodular growth pattern, characterized by multiple, unencapsulated tumor nodules dispersed within fibrous connective tissue or adjacent salivary parenchyma (Figure 1). These nodules were separated by dense fibrous septa and often located near areas of surgical fibrosis.

Compared to primary lesions, RPAs more frequently showed a reduced stromal component and relatively increased cellularity in focal areas. The stroma was predominantly fibrous or hyalinized, with myxoid material less abundant than in non‐recurrent tumors. Cystic degeneration and squamous metaplasia were identified in several nodules, particularly in association with scarred regions. No necrosis or mitotic activity was detected, and nuclear atypia was absent in all cases.

All patients underwent surgical treatment. However, information regarding the type of initial parotidectomy (superficial or total) and surgical margin status was available only for cases that were initially treated at our institution. Surgical details were retrievable in 8 cases: 7 patients (30.4%) underwent superficial parotidectomy, and 1 patient (4.3%) underwent total parotidectomy. Among these, surgical margins were reported as compromised in 5 cases (21.7%) and free in 3 cases (13.0%). Margin status was available only for cases initially treated at our institution and should therefore be interpreted with caution. For the remaining 15 patients (65.2%), margin status and type of procedure were not available, as the initial surgical management had been performed at external institutions. Follow‐up data were available for all patients, with a mean follow‐up time of 78.7 ± 53.1 months, ranging from 3.6 to 182.9 months. All patients (23; 100%) were alive and disease‐free at last follow‐up.

### 3.4 Carcinoma ex Pleomorphic Adenoma

Sixty‐three cases of CXPA were analyzed. The mean age at diagnosis was approximately 58.5 ± 13.9 years (range: 27–88 years). Among cases with available information, 35 were male (55.6%) and 23 were female (36.5%). The parotid gland was the most affected site (44 cases; 69.8%), followed by the submandibular gland (9 cases; 14.3%), and minor salivary glands (9 cases; 14.3%). The history of tobacco use was identified in 19 patients (30.2%), and alcohol consumption was noted in 12 cases (19.0%).

Clinically, symptomatology was documented in a subset of cases. Twenty tumors (31.7%) were asymptomatic at presentation, whereas six patients (9.5%) presented with symptoms, including pain (*n* = 6), dysphonia (*n* = 1), and facial paralysis associated with pain (*n* = 1). In the remaining 35 cases (55.6%), no information regarding symptomatology was available in the medical records. The clinical presentation commonly involved nodules with a fibrous consistency, observed in 32 cases (50.8%). Most lesions were mobile (12 cases; 19.0%). Tumor size ranged from 1.5 to 10.0 cm, with a mean of 5.06 ± 2.24 cm. Time from initial symptoms to diagnosis ranged from 3 to 360 months, with a mean of 98 ± 100 months. A clinical history of prior pleomorphic adenoma was confirmed in 13 patients (20.6%).

Clinical staging revealed that 19 tumors (30.2%) were classified as T1–T2 and 23 (36.5%) as T3–T4. Nodal metastasis (N+) was present in 12 cases (19.0%), with 32 (50.8%) staged as N0. Distant metastasis at diagnosis was identified in 2 cases (3.2%). When grouped, 27 cases (42.9%) were staged as stage III–IV, and 15 (23.8%) as stage I–II. All patients underwent surgical treatment. Thirty patients (47.6%) underwent surgery alone, 4 (6.3%) received surgery and neck dissection, 13 (20.6%) underwent surgery followed by radiotherapy, 9 (14.3%) surgery, neck dissection and radiotherapy, and only one (1.6%) received surgery, neck dissection, and chemotherapy.

**FIGURE 2:**
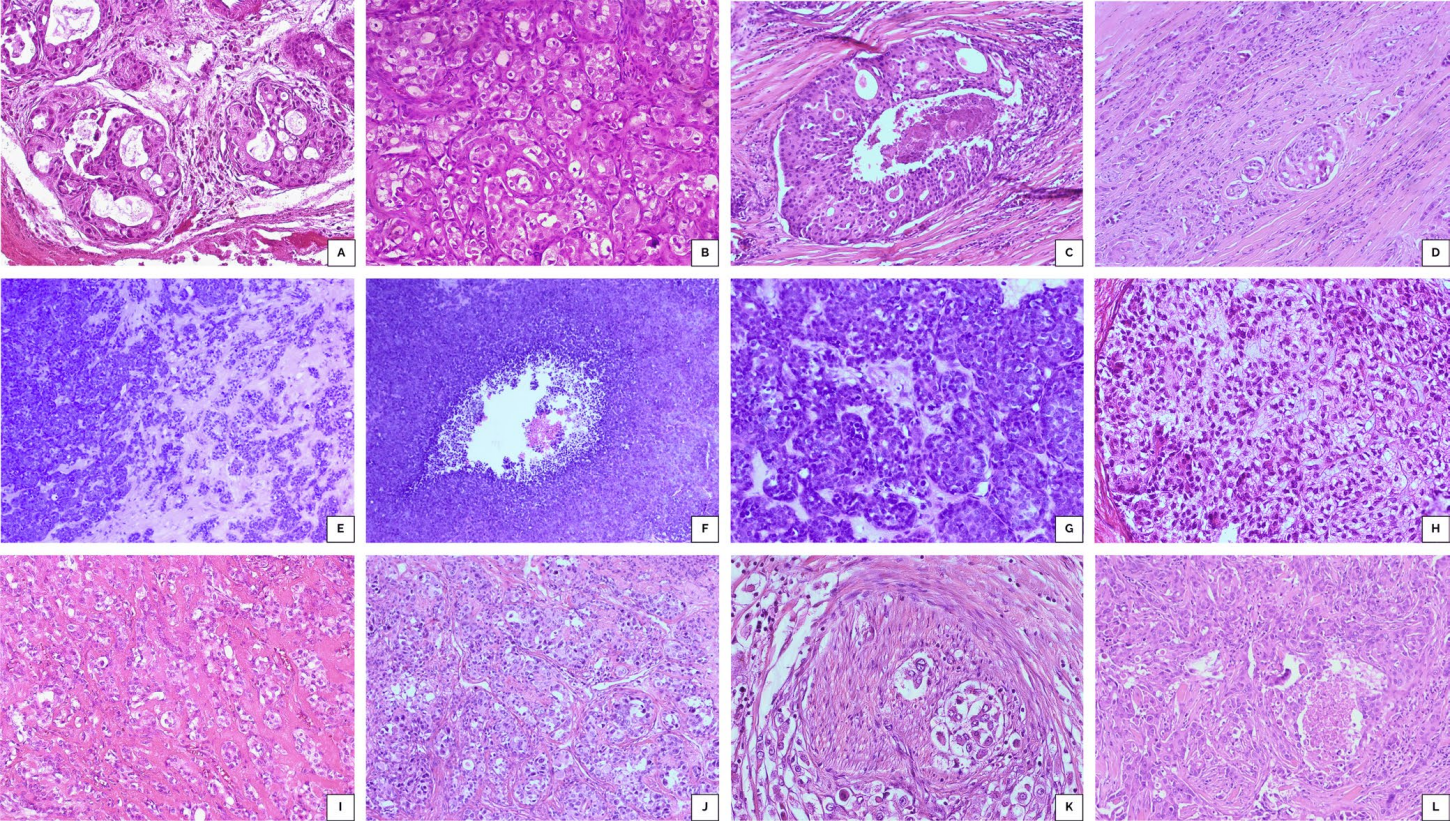
Representative histopathological features of carcinoma ex pleomorphic adenoma (CXPA) displaying distinct malignant subtypes and invasion patterns. (A–D) Salivary duct carcinoma (SDC) subtype: (A) solid epithelial nests with Roman bridge‐like structures (20×); (B) solid areas composed of large polygonal cells with eosinophilic cytoplasm, prominent nucleoli, and nuclear pleomorphism (20×); (C) cohesive epithelial proliferation forming duct‐like structures with central necrosis and Roman bridge‐like structures (10×); (D) perineural/neural invasion accompanied by deep infiltration of adjacent skeletal muscle fibers by malignant epithelial cells (10×). (E–G) Myoepithelial carcinoma subtype: (E) transition between residual pleomorphic adenoma and malignant myoepithelial proliferation (10×); (F) central necrosis surrounded by densely packed malignant cells (10×); (G) solid islands of pleomorphic myoepithelial cells with hyperchromatic, bizarre nuclei and scant cytoplasm (20×). (H, I) Epithelial–myoepithelial carcinoma subtype: Solid nests composed of an inner layer of eosinophilic epithelial cells and an outer rim of clear myoepithelial cells, arranged in lobules and separated by thick fibrous septa; tumor cells exhibit moderate nuclear atypia (20× each). (J–L) Adenocarcinoma, not otherwise specified (AdNOS) subtype: (J) high‐grade tumor with solid growth, marked nuclear pleomorphism, and prominent nucleoli (10×); (K) perineural/neural invasion by poorly differentiated tumor cells (40×); (L) extensive necrosis within a densely cellular neoplastic area (10×). All sections were stained with hematoxylin and eosin (H&E).

Histologically, CXPA demonstrated a biphasic histological profile, comprising a residual benign PA component and a malignant epithelial proliferation. The residual benign areas showed conventional features of PA, including ductal and myoepithelial elements embedded in a fibromyxoid or chondromyxoid stroma. The malignant component exhibited pronounced architectural and cytological atypia, with solid, cribriform, or infiltrative growth patterns, each varying according to the histological subtype of the neoplasm (Figure 2). Nuclear pleomorphism, prominent nucleoli, increased mitotic activity, and areas of necrosis were commonly observed. The most prevalent histological subtype was SDC (22 cases; 34.9%), followed by Adenocarcinoma, not otherwise specified (AdNOS) (20; 31.7%), MC (9; 14.3%), and epithelial‐myoepithelial carcinoma (EMC) (9; 14.3%). Less common types included epidermoid carcinoma, sarcomatoid carcinoma, secretory carcinoma, and undifferentiated carcinoma.

Based on the extent of capsular invasion, invasive tumors were the most frequent pattern, accounting for 31 cases (49.2%), followed by MI (15 cases; 23.8%) and IC (17 cases; 27.0%). Perineural invasion was observed in 8 cases (12.7%) and angiolymphatic invasion in 7 (11.1%). Surgical margins were compromised in 21 patients (33.3%) and free in 23 cases (36.5%). Margin status was therefore evaluable in 44 cases (69.8%), while it was unavailable in 19 cases (30.2%). Only cases with documented margin status were included in the survival analyses involving this variable.

**FIGURE 3:**
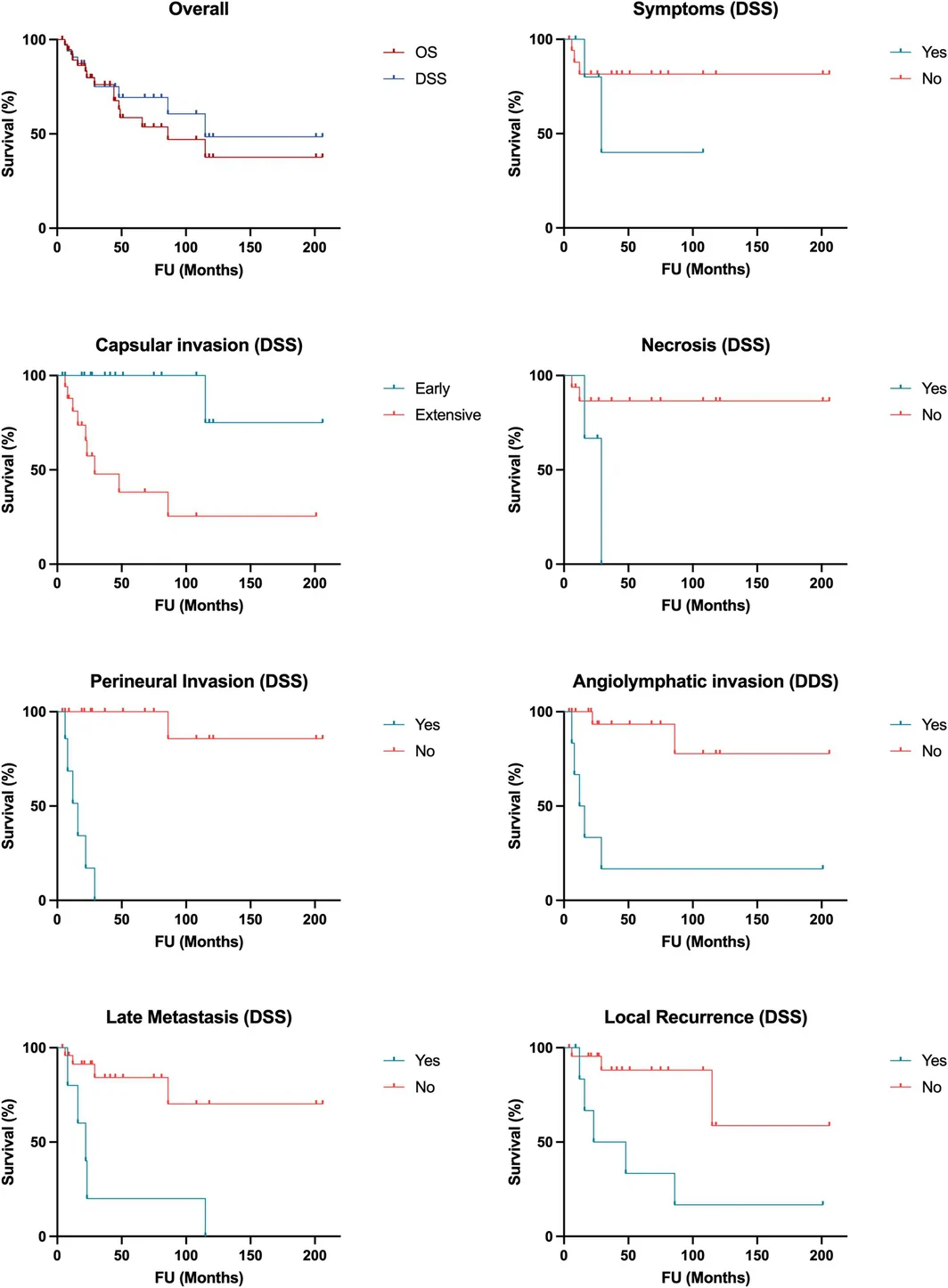
Disease‐specific survival curves for carcinoma ex pleomorphic adenoma.

Tumor recurrence occurred in 7 patients (11.1%), while 28 (44.4%) had no recurrence. Data was missing on 28. Late metastases occurred in 5 patients (7.9%) and included involvement of the orbit, lungs, vertebrae, and lymph nodes. Among the 37 patients with complete follow‐up information available for survival analysis, the mean follow‐up time was 57.6 ± 69.9 months, ranging from 0.3 to 371.9 months. The remaining 26 patients (41.3%) had incomplete or unavailable follow‐up data and were therefore excluded from the survival analyses. At last follow‐up, 21 patients (33.3%) were alive and disease‐free, 11 (17.5%) had died of disease, and 5 (7.9%) had died of other causes. The five‐year OS and DSS rates were 53.7% and 69.2%, respectively, while the ten‐year OS and DSS rates declined to 37.6% and 48.5%, respectively (Figure 3).

### 3.5 Comparative and Survival Analysis

Comparative analysis between benign (PA and RPA) and malignant (CXPA) tumors using Fisher's exact test revealed significant differences in several clinicopathological features. Patients with CXPA were significantly older than 45 years (*p* < 0.0001). Female sex was significantly more frequent in the benign group (*p* = 0.0094). Tumors smaller than 4 cm were more commonly observed in the benign group (*p* = 0.0042), and the benign group also showed significantly shorter clinical evolution times (*p* = 0.0019).

Among the benign tumors (PA vs. RPA), the Fisher's exact test showed that clinical evolution time > 45 months was significantly associated with recurrent tumors (*p* = 0.042). Patients with RPA were diagnosed at a younger estimated age at primary PA diagnosis (mean 34.6 years) compared with patients without recurrence (mean 43.7 years; *p* = 0.0232).

**FIGURE 4:**
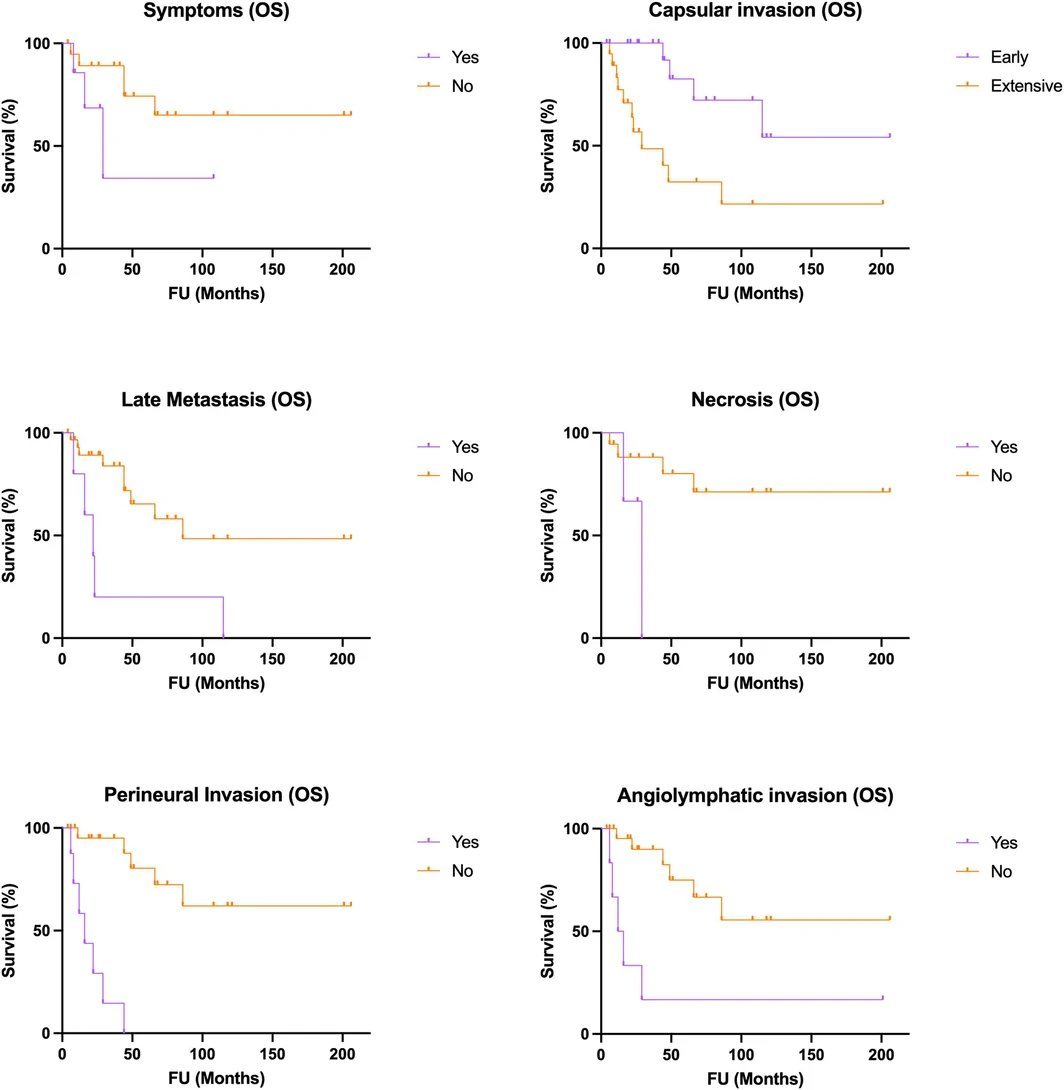
Overall survival curves for carcinoma ex pleomorphic adenoma.

Univariate Cox regression analysis revealed several clinical and pathological factors significantly associated with worse DSS and OS in CXPA (Table 2, Figure 3 and 4). For OS, adverse prognostic factors included the presence of symptomatology (*p* = 0.040), late distant metastasis (*p* = 0.009), frank capsular invasion (*p* = 0.037), necrosis (*p* = 0.017), angiolymphatic invasion (*p* = 0.0005), and perineural invasion (*p* = 0.0002) (Table 2, Figure 4). Similarly, reduced DSS was associated with perineural invasion (*p* = 0.002), angiolymphatic invasion (*p* = 0.005), necrosis (*p* = 0.026), and capsular invasion pattern (*p* = 0.010) (Table 2, Figure 3). In addition, clinical variables, including the presence of symptomatology (*p* = 0.034), recurrence (*p* = 0.035), and late distant metastasis (*p* = 0.008), were associated with reduced DSS (Table 2, Figure 3). All clinicopathological variables discussed in the text, including recurrence, distant metastasis, perineural invasion, angiolymphatic invasion, necrosis, and capsular invasion, are detailed in Table 2 with corresponding hazard ratios and 95% confidence intervals. Multivariate analysis was not conducted due to the limited number of outcome events, which precluded adequate statistical power. Accordingly, the prognostic associations identified should be interpreted within the context of univariate analyses.

**TABLE 2:** Univariate Cox proportional hazards regression evaluating the association between clinicopathological variables and overall survival and disease‐specific survival in carcinoma ex pleomorphic adenoma.

Variable	Overall survival			Disease‐specific survival		
	HR (95% CI)	*χ* ^2^	*p*	HR (95% CI)	*χ* ^2^	*p*
Sex						
Male	0.6934 (0.2190–2.1952)	0.39	0.5334	0.7682 (0.2005–2.9423)	0.15	0.7003
Female	1.4421 (0.4555–4.5655)			1.3017 (0.3398–4.9856)		
Age						
≤ 45 years	0.3335 (0.04333–2.6016)	1.09	0.2961	1.5121 (0.1904–12.0092)	0.15	0.6956
> 45 years	2.9784 (0.3844–23.0794)			0.6613 (0.0832–5.2520)		
Anatomical site						
Major salivary gland	0.7280 (0.1529–3.4649)	0.16	0.6901	0.8221 (0.1680–4.0227)	0.06	0.8089
Minor salivary gland	1.3734 (0.2886–6.5365)			1.2163 (0.2485–5.9518)		
Symptoms						
Yes	4.1467 (1.0640–16.1602)	4.2	**0.0404**	6.3457 (1.1426–35.2419)	4.46	**0.0346**
No	0.2411 (0.06188–0.9397)			0.1575 (0.0283–0.8751)		
Evolution time						
≤ 55 months	1.1461 (0.3715–3.5350)	0.06	0.8123	1.7516 (0.3596–8.5301)	0.48	0.4876
> 55 months	0.8724 (0.2828–2.6910)			0.5709 (0.1172–2.7802)		
T classification						
T1–T2	0.7139 (0.2346–2.1719)	0.35	0.5528	0.3187 (0.0667–1.5208)	2.06	0.1515
T3–T4	1.4006 (0.4604–4.2608)			3.1376 (0.6575–14.9722)		
N classification						
N−	0.4077 (0.1393–1.1930)	2.68	0.1014	0.3343 (0.0942–1.1861)	2.88	0.0899
N+	2.4526 (0.8381–7.1766)			2.9909 (0.8430–10.6117)		
Stage						
I–II	0.4497 (0.1238–1.6339)	1.47	0.2247			
III–IV	2.2233 (0.6119–8.0773)					
Treatment						
Surgery alone	0.8181 (0.2904–2.3041)	0.14	0.7039	0.1581 (0.0198–1.2641)	3.03	0.08172
Combined treatment modalities	1.2223 (0.4339–3.4427)			6.3242 (0.7927–50.4529)		
Histological pattern						
Epithelial	1.3022 (0.3666–4.6259)	0.17	0.6829	1.6629 (0.3568–7.7484)	0.42	0.5171
Epithelial + myoepithelial	0.7678 (0.2161–2.7276)			0.6013 (0.1290–2.8020)		
Capsular invasion						
Intracapsular‐ minimally	0.3186 (0.1084–0.9359)	4.33	**0.0374**	0.1315 (0.0279–0.6197)	6.58	**0.0103**
Frankly	3.1385 (1.0684–9.2196)			7.6005 (1.6135–35.8014)		
Necrosis						
Present	9.0261 (1.4638–55.65543)	5.62	**0.0177**	7.8201 (1.2718–48.0836)	4.93	**0.0264**
Absent	0.1107 (0.0179–0.6831)			0.1278 (0.0207–0.7862)		
Perineural invasion						
Present	23.2505 (4.3983to 122.9072)	13.72	**0.0002**	28.3675 (3.2869–244.8211)	9.25	**0.0023**
Absent	0.04301 (0.0081–0.2273)			0.0352 (0.0040–0.3042)		
Angiolymphatic invasion						
Present	5.6560 (1.6843–18.9925)	7.86	**0.0005**	7.5718 (1.7869–32.0852)	7.55	**0.0059**
Absent	0.1768 (0.0526–0.5936)			0.1320 (0.0311–0.5596)		
Surgical margins						
Negative	0.6662 (0.2326–1.9076)	0.57	0.4492	0.6910 (0.1990–2.3992)	0.34	0.5605
Positive	1.5009 (0.5241–4.2975)			1.4471 (0.4167–5.0245)		
Local recurrence						
Present	2.5536 (0.8297–7.8589)	2.67	0.1021	4.1292 (1.104121–15.44307)	4.44	**0.0351**
Absent	0.2421 (0.0647–0.9056)			0.2421 (0.0647–0.9056)		
Metastasis						
Present	4.4216 (1.4484–13.4974)	6.82	**0.0090**	5.4244 (1.5385–19.12406)	6.92	**0.0085**
Absent	0.2261 (0.0740–0.6903)			0.1843 (0.05229–0.6499)		

*Note:* The significant of bold value is < 0.05 (*p*‐value < 0.05).

Abbreviations: CI, confidence interval; HR, hazard ratio.

## 4 Discussion

This study provides a comparative analysis of PA, RPA, and CXPA, focusing on their clinicopathological features and prognostic factors. By evaluating these entities side by side, we aimed to identify patterns that may support risk stratification and inform clinical management. This analysis contributes to the limited literature on salivary gland tumors and provides comprehensive data from a large single‐institution cohort.

Our results corroborate the well‐established epidemiological profile of PA as one of the most prevalent salivary gland neoplasms, predominantly affecting middle‐aged women and primarily involving the parotid gland. These observations are consistent with previous reports indicating a female predominance and peak incidence between the third and fifth decades of life (de Oliveira et al. 2009; Tian et al. 2010; Lingam et al. 2011; Andreasen et al. 2016; de Sousa Lopes et al. 2017). Furthermore, a comparative analysis of RPA cases within our cohort revealed comparable sex distribution and age at recurrence, as reported in other studies (Laskawi et al. 1998; Abdallah et al. 2024). It is noteworthy that when the age at initial PA diagnosis was estimated in patients who subsequently experienced recurrence, a lower mean age was observed compared with those patients without recurrence (34.6 vs. 43.7 years, respectively). This finding is consistent with the conclusions of earlier studies, which indicated that a younger age at first presentation may be associated with an increased risk of recurrence (McGregor et al. 1988; Laskawi et al. 1998; Wittekindt et al. 2007; Abu‐Ghanem et al. 2016; Dulguerov et al. 2017). While this association has not been consistently demonstrated in all series (Riad et al. 2011), the present data support the hypothesis that younger age may represent a potential risk factor for recurrence in PA.

Regarding CXPA and sex predilection, the findings of the present study are not in line with the conclusions of previously published studies. The studies demonstrated a preponderance of female subjects (Olsen and Lewis 2001; Katabi et al. 2010). However, the predominance of male patients, as observed in the present study, has also been reported by other authors (Olsen and Lewis 2001; Liu et al. 2023). As expected, CXPA cases predominantly affected the elderly population, with a mean age of approximately 60 years, in agreement with prior studies (Olsen and Lewis 2001; Katabi et al. 2010; Suzuki et al. 2016; Liu et al. 2023). When the mean age of CXPA was compared with patients with PA, the mean age was found to be significantly higher by approximately 15 years. This discrepancy has been documented in previous studies that have compared the clinical features of PA and CXPA (Seok et al. 2019). Even though both our analysis and that of Seok et al. found this age difference to be statistically significant, it is important to note that CXPA can still occur in younger patients, as was observed in our series, where some cases were diagnosed before the age of 45 (Seok et al. 2019).

In addition to an analysis of demographic patterns, a comprehensive evaluation of key clinical parameters was also conducted to achieve a more detailed characterization of the behavior and presentation of PA, RPA, and CXPA. Regarding the clinical presentation, the location of the tumor followed the expected anatomical patterns across all groups. The parotid gland was the most affected site in PA (80.4%), RPA (100%), and CXPA (69.8%) cases, with no substantial differences observed between groups. While the involvement of the submandibular and minor salivary glands was occasionally noted in PA and CXPA, RPA cases were exclusively parotid in origin. This finding serves to reinforce previous evidence that recurrences predominantly arise from parotid tumors, possibly due to the gland's complex anatomy and surgical challenges (Henriksson et al. 1998; Dulguerov et al. 2017). Although our study did not demonstrate an association between tumor site and survival, previous research suggests that anatomical location may influence clinical outcomes in salivary gland tumors (dos Santos et al. 2021).

Typically, the PA presents as a solitary, painless and slow‐growing tumor (de Sousa Lopes et al. 2017). Recurrent PA generally retains the clinical features of the primary lesion, often presenting as multiple painless, well‐circumscribed nodules. Within the confines of our cohort, no statistically significant discrepancy in tumor size was observed between cases of PA and RPA. Although there are studies indicating a propensity for recurrence in larger tumors (Ghosh et al. 2003; Riad et al. 2011), our findings align with those of Henriksson et al. (1998), who reported an absence of a clear association between tumor size and recurrence. While clinical tumor size is a relevant factor in surgical planning and risk evaluation, recurrence may be more directly influenced by the anatomical complexity of the region and the technical difficulty in achieving complete resection. Therefore, while tumor size may contribute to recurrence in selected cases, it does not appear to be a consistent or independent predictor.

In contrast, CXPA tumors in our cohort exhibited significantly larger dimensions than their benign counterparts, and this discrepancy was found to be statistically significant. This observation is corroborated by several studies, which have associated larger tumor size with the development of CXPA (Seok et al. 2019; Yin et al. 2021; Levyn et al. 2023). Tumor enlargement in CXPA is frequently regarded as an indirect indicator of malignant transformation, especially when associated with rapid growth or atypical clinical features, such as pain or facial nerve involvement. However, tumor size may also reflect the duration of tumor evolution prior to diagnosis, rather than aggressiveness alone, a factor further explored in our analysis of clinical evolution time.

Notably, although a relatively high proportion of compromised surgical margins was observed in PA, the documented recurrence rate during the available follow‐up was low. This finding should be interpreted with caution. PA is an indolent tumor, and recurrences may occur many years after initial surgery, potentially beyond the follow‐up period captured in retrospective analyses. Moreover, as this study was conducted in a tertiary referral hospital, patients with benign disease are frequently discharged from specialized follow‐up after initial postoperative care and may continue monitoring elsewhere or return only if symptoms recur. This referral‐based follow‐up pattern may contribute to an underestimation of late recurrences, particularly in cases with close or compromised margins.

In addition to tumor size and margin status, the duration of symptoms prior to diagnosis exhibited significant variation across different tumor types. In our cohort, RPA cases, representing a distinct group of patients with clinically confirmed recurrence of previously diagnosed PA, exhibited a significantly longer mean clinical evolution time compared with non‐recurrent PA cases. A clinical evolution time exceeding 45 months was found to be significantly associated with recurrence among benign tumors (*p* = 0.042). This association may be multifactorial. Patients with a prior history of PA may delay seeking care when recurrence occurs, owing to their awareness of the previous benign nature of the tumor and its characteristically indolent behavior. Furthermore, recurrent lesions often present as slow‐growing, multifocal nodules, which may contribute to delayed clinical suspicion and diagnosis. Although a prolonged duration of symptoms was statistically associated with recurrence in our cohort, this should be interpreted as a clinical correlation rather than a direct causal factor. Nonetheless, these findings underscore the importance of long‐term clinical follow‐up in patients with a history of PA, even after initial successful treatment.

Our results also demonstrated that PA and RPA cases had significantly shorter clinical courses when compared with CXPA, reinforcing the notion that diagnostic delay may contribute to the larger size observed in malignant tumors. Seok et al. (2019) reported findings similar to those of the present study. They also noted that patients with CXPA experienced a significantly longer time to diagnosis than those with benign PA (Seok et al. 2019). These findings underscore the importance of careful monitoring of persistent or slow‐growing salivary gland lesions, especially in older patients or when atypical features emerge over time.

Histologically, SDC was the most frequently identified malignant subtype within CXPA cases, followed by AdNOS, and MC. This distribution is consistent with prior reports and reflects the aggressive biological behavior typically attributed to these entities (Katabi et al. 2010; Mariano et al. 2013; Lim et al. 2015; Suzuki et al. 2016). However, in our series, histological subtypes were not significantly associated with overall survival. This finding aligns with the results reported by Zhao et al. (2013) and Olsen and Lewis (2001), who also found no statistical correlation between histological subtype and prognosis in CXPA (Olsen and Lewis 2001; Zhao et al. 2013). Frankly capsular invasion was the most common pattern of invasion, and it was significantly associated with poorer DSS in our series. These findings are consistent with many studies, which emphasize the prognostic relevance of the extent of invasion (Brandwein et al. 1996; Lewis et al. 2001; Olsen and Lewis 2001; Katabi et al. 2010; Zhao et al. 2013; Lim et al. 2015; Rito and Fonseca 2016; Ye et al. 2016; Hu et al. 2020). However, as Katabi et al. (2010) observed, the absence or minimal extent of invasion does not ensure favorable outcomes (Katabi et al. 2010). This assertion is corroborated by the observation that two patients in our cohort, who were classified as intracapsular or minimally invasive, died of the disease, underscoring the implication that a minimal degree of invasion may not necessarily preclude an aggressive clinical course.

Several histopathological features traditionally associated with aggressive behavior were confirmed as significant prognostic indicators in our univariate survival analysis. Perineural invasion, angiolymphatic invasion, and necrosis were all significantly associated with reduced OS and DSS. These features are well‐established markers of high‐grade malignancy and have been consistently reported as predictors of poor outcome in CXPA (Katabi et al. 2010; Hu et al. 2016, 2020; Ma et al. 2021; Liu et al. 2023). Additionally, the presence of recurrence and late distant metastases had a significant impact on both OS and DSS, highlighting the impact of advanced disease on prognosis (Livolsi and Perzin 1977; Chen et al. 2007, 2014; Lüers et al. 2009; Katabi et al. 2010; Zhao et al. 2013; Xiao et al. 2016). In the present cohort, local recurrence was observed in 11.3% of patients, while 7.9% developed delayed distant metastases involving sites such as the orbit, lung, vertebrae, and lymph nodes.

From a clinical standpoint, the findings of this study emphasize the need for prolonged and individualized follow‐up strategies in patients with PA, particularly in younger individuals and in cases with long‐standing clinical evolution, given their association with recurrence in our cohort. Long‐term surveillance of persistent salivary gland lesions is especially relevant, as diagnostic delay may contribute not only to increased tumor size but also to malignant transformation. Moreover, the strong prognostic impact of perineural invasion, angiolymphatic invasion, necrosis, and the extent of capsular invasion in CXPA underscores the critical role of comprehensive histopathological evaluation. Systematic reporting of these features in pathology reports is essential to support risk stratification, guide therapeutic decision‐making, and inform follow‐up planning in clinical practice.

This study presents several limitations inherent to its retrospective design. The PA, RPA, and CXPA cases analyzed represent distinct groups and were not derived from a single longitudinal cohort that progressed from benign to malignant forms. Therefore, the associations reported are based on cross‐sectional comparisons rather than direct disease evolution within individual patients. This distinction is critical when interpreting the absence of malignant transformation observed in our PA and RPA cohorts. Such findings may also reflect limited long‐term follow‐up in benign cases, where clinical surveillance is often discontinued, and patients typically return only if symptoms recur, potentially delaying the detection of late malignant events.

In addition, as this study was conducted at a single tertiary referral center, a potential selection bias must be considered, as more complex or advanced cases may be over‐represented. Additional limitations include missing data in medical records, variability in treatment strategies across decades, and incomplete follow‐up in a substantial proportion of CXPA cases, with approximately one‐third of patients lacking adequate follow‐up for survival analysis. Together, these factors may contribute to an underestimation of malignant transformation of PA, particularly in patients discharged after apparently curative surgery. These factors should be considered when interpreting the prognostic findings. Nevertheless, the study's strengths include a robust sample size, meticulous clinicopathological review, and comprehensive statistical evaluation.

## 5 Conclusion

In conclusion, this 20‐year retrospective analysis offers a comprehensive characterization of PA, RPA, and CXPA, underscoring their distinct clinicopathological profiles and prognostic behaviors. Tumor size, symptom duration, perineural and angiolymphatic invasion, necrosis, extent of capsular invasion, and metastatic disease were identified as relevant factors for clinical decision‐making, risk stratification, and long‐term follow‐up. These results enhance existing knowledge and add valuable epidemiological data from a Latin American cohort, contributing to future clinical guidelines for salivary gland tumor management.

## Author Contributions

**Talita de Carvalho Kimura:** conceptualization, investigation, writing – original draft, methodology, writing – review and editing, formal analysis, data curation. **Luccas Lavareze:** methodology, writing – review and editing, formal analysis, data curation. **Reydson Alcides de Lima‐Souza:** writing – review and editing, formal analysis. **Nívia Castro Binda:** writing – review and editing, formal analysis. **Carlos Takahiro Chone:** writing – review and editing. **Alfio José Tincani:** writing – review and editing. **Albina Altemani:** writing – review and editing. **Fernanda Viviane Mariano:** conceptualization, investigation, writing – review and editing, supervision.

## Ethics Statement

This study was approved by the Research Ethics Committee of the State University of Campinas (UNICAMP) (CAAE: 59408016.7.0000.5404 and CAAE: 59408016.7.3001.5418).

## Conflicts of Interest

The authors declare no conflicts of interest.

## Data Availability

The authors confirm that the data supporting the findings of this study are available within the article.
